# Carbolate of Iodine

**Published:** 1867-12

**Authors:** D'Oyley Evans

**Affiliations:** Paris, France.


### ARTICLE VI.

### *Carbolate of Iodine.*

By D'Oyley Evans, D.D.S., Paris, France.

Messrs. Editors:—

I have just read a letter published in *Le Journal des Connaissances Medicales*, addressed to Dr. Caffe, on Dr. Percy Boulton's late discovery of the action of Carbolic or Phenic acid on Iodine.

And thinking that after the conversation I had with you while in America about Phenic acid, that the above mentioned letter may interest you, I will give it, with some observations of my own apropos to the same combination, which I have used for some time for inflammations of the mouth.

"The inconvenience," says the writer, "attending the external application of iodine and its preparations is so serious, that physicians are often obliged to abandon a remedy, the therapeutic efficacy of which is undoubted, nay almost unequalled in materia medica." "The great objection to the external use of this remedy is, that it leaves marks both on the linen and on the skin."

"This is a sufficient motive for seeking some means of getting rid of this drawback, especially in the case of ladies." Dr. Percy Boulton's method consists in adding a few drops of Phenic, (carbolic) acid to the iodine solution to be employed." "This addition renders the iodine perfectly colorless, so that it may be applied with impunity." But this combination has another advantage. It appears from that practitioner's observations, which I can confirm, that, so administered, *Carbolate of Iodine,* which is the new substance in question, is not only one of the most powerful antiseptics we possess, but is intrinsically a more efficacious agent than iodine alone. I have used this compound in the form of injections, gargles and lotions in all cases in which iodine is prescribed. For diseases of the antrum, abscess, sore-throat and inflammation of the gums.

This preparation is almost a sovereign remedy, since besides its disinfecting qualities, it modifies the mucous membrane, causes all local sensibility to disappear, and cures the patient much sooner than if either of the two agents were employed separately. The formula I employ is as follows:

| | | |
|---|---|---|
| ℞. | Compound Tinct. of Iodine . . | 3 gms. |
| | Pure Carbolic acid . . . | 6 gtts. |
| | Glycerin . . . . . | 30 gms. |
| | Distilled Water . . . | 150 gms. |

